# Supplementary figures and images for: Effects of intraoperative hypothermia on patients undergoing laparoscopic surgery: A retrospective cohort study
Source: PLoS One. 2025 Jan 30;20(1):e0314968. doi: 10.1371/journal.pone.0314968 (PMC11781613; doi:10.1371/journal.pone.0314968)

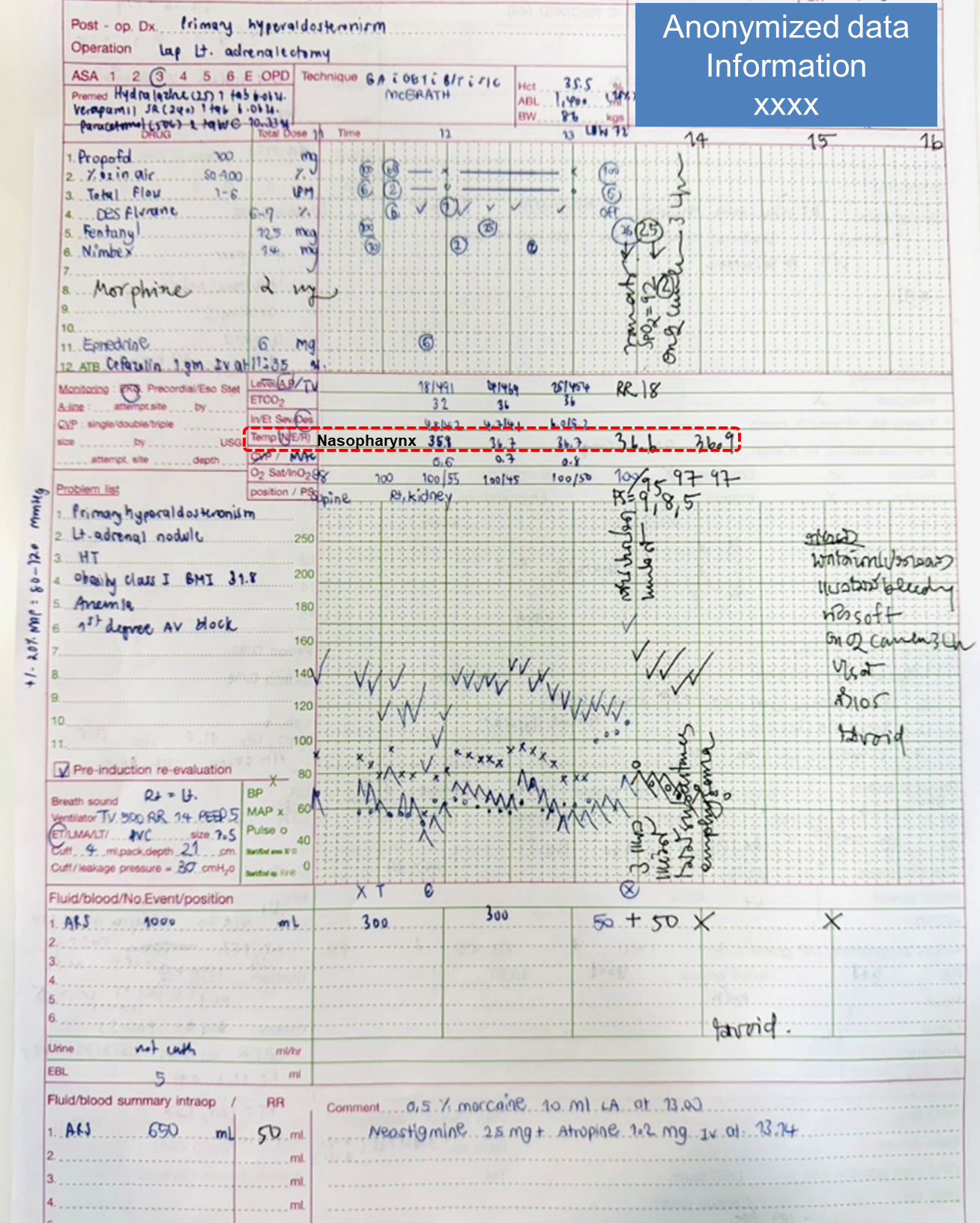

Supplement: S1 Fig — (TIF) [file pone.0314968.s001.tif]

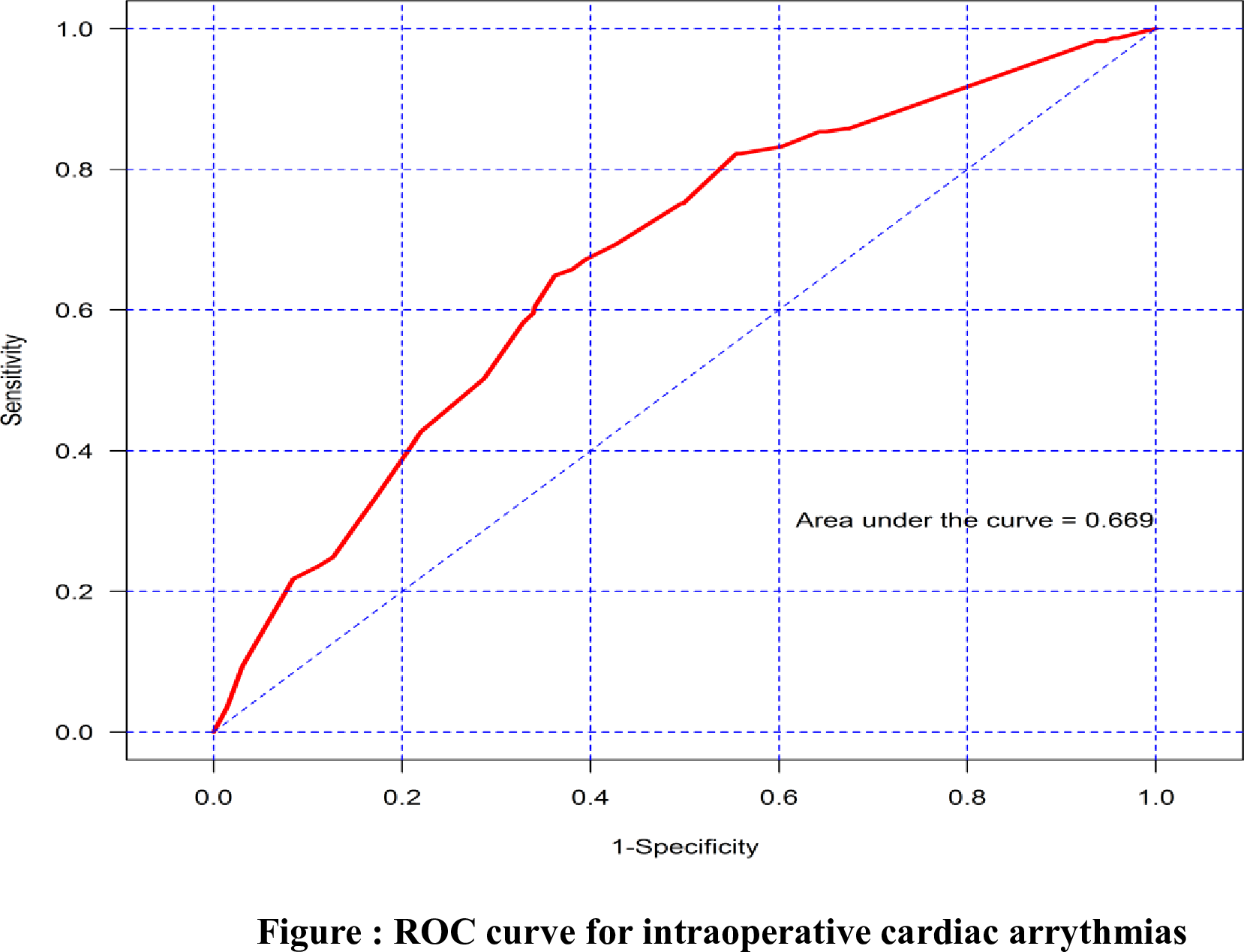

Supplement: S2 Fig — (TIF) [file pone.0314968.s002.tif]

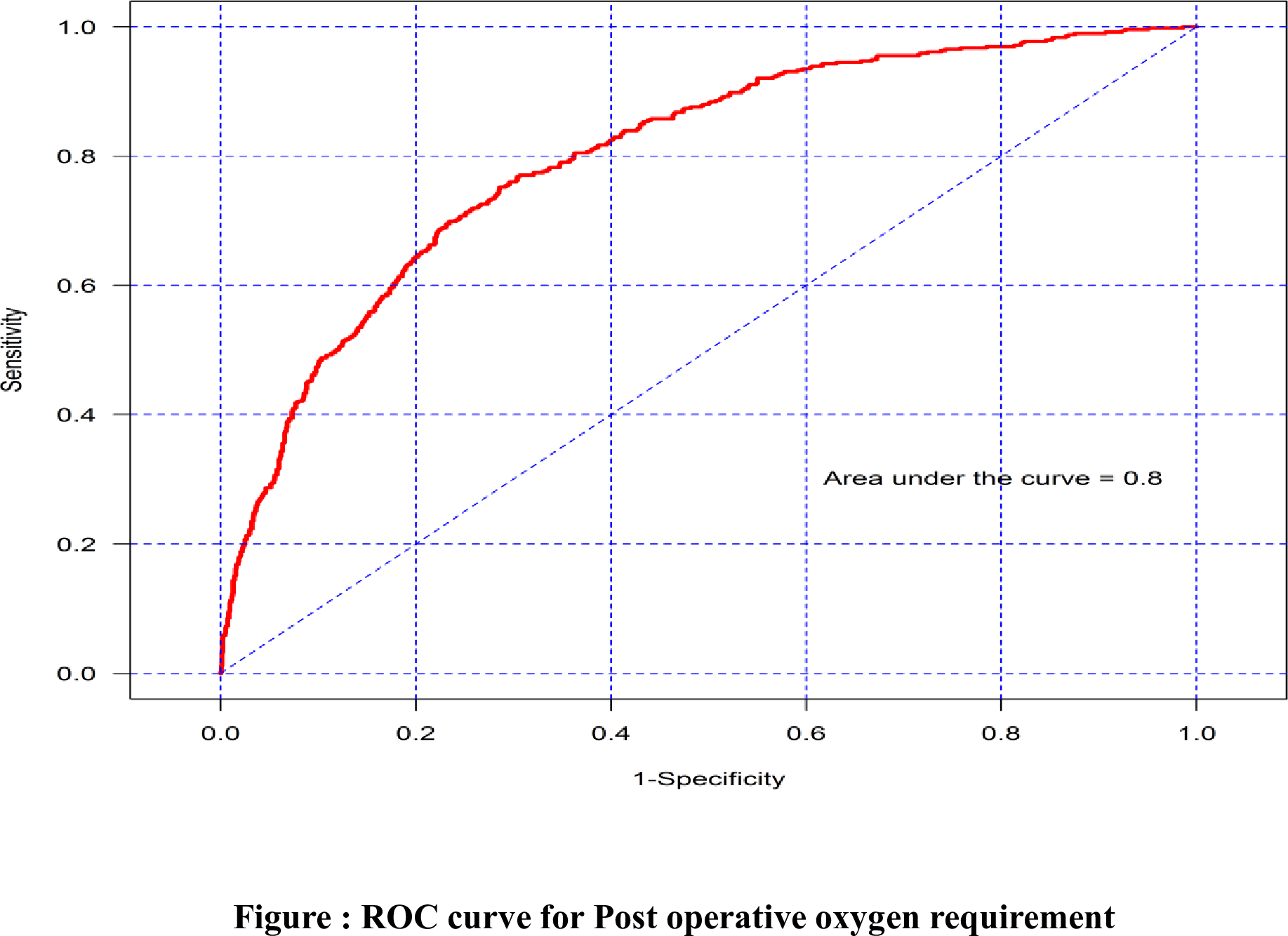

Supplement: S3 Fig — (TIF) [file pone.0314968.s003.tif]
