# Supplementary material for: Effects of intraoperative hypothermia on patients undergoing laparoscopic surgery: A retrospective cohort study
Source: PLoS One. 2025 Jan 30;20(1):e0314968. doi: 10.1371/journal.pone.0314968 (PMC11781613; doi:10.1371/journal.pone.0314968)
Supplement: S1 Table — (DOCX) [file pone.0314968.s004.docx]

**S1 Table.** Outcome comparison between normothermia and hypothermia

|  | **Normothermia** | **Hypothermia** | **P value** |
| --- | --- | --- | --- |
| **Total** | 1333 | 715 |  |
| **Intraoperative cardiac arrhythmia** |  |  | < 0.001 |
| no | 1224 (91.8) | 599 (83.8) |  |
| yes | 109 (8.2) | 116 (16.2) |  |
| **Intraoperative blood transfusion** |  |  | 0.172 |
| no | 1301 (97.5) | 688 (96.4) |  |
| yes | 33 (2.5) | 26 (3.6) |  |
| **Volume of intraoperative blood transfusion (ml),** median (IQR) | 0 (0,0) | 0 (0,0) | 0.533 |
| **PACU stay** |  |  | 0.015 |
| no | 10 (0.8) | 15 (2.1) |  |
| yes | 1323 (99.2) | 700 (97.9) |  |
| **Duration of PACU stay** |  |  | < 0.001 |
| **median (IQR)** | 45 (35,60) | 50 (40,65) |  |
| **Delayed awakening** |  |  | 0.402 |
| no | 1330 (99.8) | 711 (99.4) |  |
| yes | 3 (0.2) | 4 (0.6) |  |
| **Shivering** |  |  | 0.89 |
| no | 1304 (97.8) | 698 (97.6) |  |
| yes | 29 (2.2) | 17 (2.4) |  |
| **Nausea/vomiting** |  |  | 0.548 |
| no | 1184 (88.7) | 641 (89.7) |  |
| yes | 151 (11.3) | 74 (10.3) |  |
| **Postoperative oxygen requirement** |  |  | 0.643 |
| no | 1008 (75.6) | 548 (76.6) |  |
| yes | 325 (24.4) | 167 (23.4) |  |
| **If oxygen required, Duration of oxygen requirement (hrs.)** |  |  | 0.375 |
| **median (IQR)** | 15 (12,18) | 16(12,18) |  |
| **Type of ventilator** |  |  | 0.003 |
| ETT | 12 (0.9) | 17 (2.4) |  |
| NIPPV | 25 (1.9) | 5 (0.7) |  |
| no | 1296 (97.2) | 693 (96.9) |  |
| **Postoperative ICU** |  |  | 0.035 |
| no | 1320 (99) | 699 (97.8) |  |
| yes | 13 (1) | 16 (2.2) |  |
| **Duration of ICU stay (days)** |  |  | 0.023 |
| **median (IQR)** | 1 (1,2) | 3 (2,4) |  |
| **Duration of hospital stay (days)** |  |  | < 0.001 |
| **median (IQR)** | 4 (3,5) | 4 (3,7) |  |
| **Postoperative cardiac arrhythmia** |  |  | 0.913 |
| no | 1331 (99.8) | 713 (99.7) |  |
| yes | 2 (0.2) | 2 (0.3) |  |

**Notes:** Data are presented as frequency (%) or median (interquartile range [IQR]) unless stated otherwise. ICU= Intensive care unit, NIPPV=Non-invasive positive pressure ventilation, PACU=Post anesthetic care unit, ETT=endotracheal tube intubation.
