# Supplementary material for: Effects of intraoperative hypothermia on patients undergoing laparoscopic surgery: A retrospective cohort study
Source: PLoS One. 2025 Jan 30;20(1):e0314968. doi: 10.1371/journal.pone.0314968 (PMC11781613; doi:10.1371/journal.pone.0314968)
Supplement: Attachment — Submitted filename: response to reviewer v4.docx [file pone.0314968.s006.docx]

**Department of Anesthesiology**

Faculty of Medicine,

Prince of Songkla University, Hat Yai

Songkhla province, 90110 THAILAND

Tel. +66 74 429621

Fax +66 74 212900, 212903

E-mail: oomaliwa@gmail.com

8 Oct 2024

Dear PLOS ONE Editor,

We would like to resubmit a manuscript entitled “**Effects of Intraoperative Hypothermia on Patients Undergoing Laparoscopic Surgery: A Retrospective Cohort Study**” as an original article in your journal.

We are thankful to have a chance to revise our manuscript. To address the editor’s comment, we have added the information in the Tables and discussion section, updated the reference, and deposited our protocol in [protocols.io](http://protocols.io/). We also highlighted the changes in the current version. Attached is a point-by-point response to the reviewer’s concerns.

Comments from the Editors

When submitting your revision, we need you to address these additional requirements.

1. Please ensure that your manuscript meets PLOS ONE's style requirements, including those for file naming. The PLOS ONE style templates can be found at

https://journals.plos.org/plosone/s/file?id=wjVg/PLOSOne_formatting_sample_main_body.pdf and

<https://journals.plos.org/plosone/s/file?id=ba62/PLOSOne_formatting_sample_title_authors_affiliations.pdf>

**Response**: We ensure that our manuscript meets PLOS ONE's style requirements, including those for file naming.

2. Thank you for stating the following financial disclosure: The funding was granted for Dr. Tashi Penjore, amount of 44,600 baht (#65-226-8-9) by the Faculty of Medicine, Prince of Songkla University.

Please state what role the funders took in the study. If the funders had no role, please state: ""The funders had no role in study design, data collection and analysis, decision to publish, or preparation of the manuscript.""

If this statement is not correct you must amend it as needed.

Please include this amended Role of Funder statement in your cover letter; we will change the online submission form on your behalf.

**Response**: The funder had no role in study design, data collection and analysis, decision to publish, or preparation of the manuscript. We include this amended Role of Funder statement in our recent cover letter.

Additional Editor Comments:

The paper is well-written and structured. In order to be evaluated for publication, changes must be made according to the reviewers' requests.

**Response**: Thank you.

Reviewers' comments:

Comments to the Author

Please use the space provided to explain your answers to the questions above. You may also include additional comments for the author, including concerns about dual publication, research ethics, or publication ethics. (Please upload your review as an attachment if it exceeds 20,000 characters)

**Reviewer #1:**

This is a well written retrospective review of patients undergoing laparoscopic surgery and experience hypothermia. The sample size is considerable and the analysis was sound.

Technically, retrospective review should not be called cohort study as those are typically prospective. The definitions otherwise are quite clear and allows repeatability of this study. The incidence of hypothermia was just over a third of the patients which is quite significant. Can the author provide reasons why did happen given it is a major element in the Surgical Care bundle and part of patient setup?

**Response:** It was a retrospective cohort study since we can determine the cause-and-effect relationship between the exposure and outcomes. In this case, the hypothermia/episode of hypothermia was an exposure, and the effects were the consequences of the hypothermia (postoperative oxygen requirement and longer PACU stay). The term “retrospective review” could not determine the cause-and-effect relationship.

The incidence of hypothermia in our study is quite high at 34.9% but it is still within the normal range found. The reasons for this high incidence could be due to the lower room temperature inside the operating rooms in our institution (18-20˚C), longer duration of surgery resulting in exposing surgical sites for a long time. Our previous protocol was as follow. Forced air warming devices are applied above the patient in every case and use of warm irrigation and warm intravenous fluid. In case the body temperature turns lower than 36 ˚C, the operating room’s temperature could be adjusted to 22-24 ˚C case by case. Yet, there was still not adequately proper warming throughout the operation. Thus, the more proper guideline mentioned in the “Implication of the study” subsection in the discussion section must be implemented (Lines 329-339).

Can the author provide insights on how this can be prevented and that high percentage be brought down? Would adding it to the checklist help? How does this figures compare to international literature? It would hugely beneficial if the authors provide insights to how it happens and recommendations on how to prevent it.

**Response**: The literature mentions the incidence of hypothermia in laparoscopic surgery to be around 29-90%. So our incidence of 34.9% still falls within that range. We think this can be prevented, as we mentioned the more proper guideline in the “Implication of the study” subsection in the discussion section must be implemented (Lines 329-339) “At the preoperative period, proper preoperative warming for at least 30 minutes should be considered in high-risk patients as found previously; however, one should be aware that the risk of burns and thermal injuries may increase. At the intraoperative period, continuously monitoring and maintaining core body temperature above 36˚C must be performed by various methods such as forced air warming devices applied under or above the patient, optimal operating room temperature, warm intravenous fluids, limiting the exposure time of surgical sites, and using warmed, humidified insufflation gases. In the PACU period, continuous warming strategies should still be considered if the core temperature remains below 36 ˚C to minimize possible adverse events. All staff should be trained on management protocols and made aware of their importance and the possible complication of aggressive warming.”

Having a checklist could also help, as one will notice the baseline temperature and will be able to perform the active interventions on time if there is hypothermia.

**Reviewer #2:**

This study explores the impact of intraoperative hypothermia on patients undergoing laparoscopic surgery, which is clinically innovative and of great significance. The research method is a retrospective cohort study with a large and comprehensive sample size, which is reliable. The setting of independent and dependent variables is reasonable and comprehensive, the statistical methods are reasonable, logistic regression and linear regression are used to determine the relationship, and there is also a clear flowchart. But there are also some shortcomings:

1.It is recommended to replace the reference with the latest one as it has a relatively long history.

**Response:** We add the two more update references (16 and 34).

2.The conclusion section can provide a clearer indication of the specific direction and focus of future research.

**Response:** We edit the conclusion on the matter (Lines 368-371).

3.In table4, table5, and table6, abbreviations for some variables (such as OR, CI, etc.) should be given their full names and explanations when they first appear to facilitate readers' understanding. For all tables, it is recommended to add more statistical information, such as the specific calculation method of P-value and the confidence level of the confidence interval.

**Response**: Thank you for the advice. We add the full name of OR (odds ratio)/ β (Beta-coefficient) and CI (confidence interval) in the legend of Table 4-6. The 95% CI, the wald p-value for each subgroup of the variable (Table 4-6) and p-value by the likelihood ratio (LR) test for categorical outcome (Table 4,5) and p-value by F test statistic for continuous outcome (Table 6) were also mentioned in the heading of each table.

We uploaded our figure files to the Preflight Analysis and Conversion Engine (PACE) digital diagnostic tool and already got the PACE corrected files for resubmission. Thank you so much for your advice.

Should you have more comments, please don’t hesitate to contact us

Thank you for your attention to our paper

Sincerely yours,

Dr. Tashi Penjore

Assoc. Prof. Maliwan Oofuvong

Assist. Prof. Sunisa Chatmongkolchart

Assist. Prof. Chanatthee Kitisiripant

Rongrung Rueangchira-urai

Jaranya Leeratiwong
