# Supplementary material for: Effects of intraoperative hypothermia on patients undergoing laparoscopic surgery: A retrospective cohort study
Source: PLoS One. 2025 Jan 30;20(1):e0314968. doi: 10.1371/journal.pone.0314968 (PMC11781613; doi:10.1371/journal.pone.0314968)
Supplement: Attachment — Submitted filename: Response to reviewer round 2.docx [file pone.0314968.s007.docx]

**Department of Anesthesiology**

Faculty of Medicine,

Prince of Songkla University, Hat Yai

Songkhla province, 90110 THAILAND

Tel. +66 74 429621

Fax +66 74 212900, 212903

E-mail: oomaliwa@gmail.com

16 Nov 2024

Dear PLOS ONE Editor,

We would like to resubmit a manuscript entitled “**Effects of Intraoperative Hypothermia on Patients Undergoing Laparoscopic Surgery: A Retrospective Cohort Study**” as an original article in your journal.

We are thankful to have a chance to revise our manuscript. To address the editor’s comment, we have added more information in the result, Table 1, S1 Table and Table 3 and discussion section. We also highlighted the changes in the current version. Attached is a point-by-point response to the reviewer’s concerns.

Journal Requirements:

Please review your reference list to ensure that it is complete and correct. If you have cited papers that have been retracted, please include the rationale for doing so in the manuscript text, or remove these references and replace them with relevant current references. Any changes to the reference list should be mentioned in the rebuttal letter that accompanies your revised manuscript. If you need to cite a retracted article, indicate the article’s retracted status in the References list and also include a citation and full reference for the retraction notice.

**Answer:** We already checked all cited references. There is no retracted article since the full articles of all cited references could be accessed.

**Comments to the Author**

1. If the authors have adequately addressed your comments raised in a previous round of review and you feel that this manuscript is now acceptable for publication, you may indicate that here to bypass the “Comments to the Author” section, enter your conflict of interest statement in the “Confidential to Editor” section, and submit your "Accept" recommendation.

Reviewer #2: (No Response)

2. Is the manuscript technically sound, and do the data support the conclusions?

The manuscript must describe a technically sound piece of scientific research with data that supports the conclusions. Experiments must have been conducted rigorously, with appropriate controls, replication, and sample sizes. The conclusions must be drawn appropriately based on the data presented.

Reviewer #2: Yes

3. Has the statistical analysis been performed appropriately and rigorously?

Reviewer #2: Yes

4. Have the authors made all data underlying the findings in their manuscript fully available?

The [PLOS Data policy](http://track.editorialmanager.com/CL0/http:%2F%2Fwww.plosone.org%2Fstatic%2Fpolicies.action%23sharing/1/010f01931b182552-91e9c1a8-5ddb-4cd4-9eff-2c095b890a35-000000/kelHISQrVZokzinpzeNfHDtJJ1dPFfDGrhHYj24ZUls=184) requires authors to make all data underlying the findings described in their manuscript fully available without restriction, with rare exception (please refer to the Data Availability Statement in the manuscript PDF file). The data should be provided as part of the manuscript or its supporting information, or deposited to a public repository. For example, in addition to summary statistics, the data points behind means, medians and variance measures should be available. If there are restrictions on publicly sharing data—e.g. participant privacy or use of data from a third party—those must be specified.

Reviewer #2: Yes

5. Is the manuscript presented in an intelligible fashion and written in standard English?

PLOS ONE does not copyedit accepted manuscripts, so the language in submitted articles must be clear, correct, and unambiguous. Any typographical or grammatical errors should be corrected at revision, so please note any specific errors here.

Reviewer #2: Yes

6. Review Comments to the Author

Please use the space provided to explain your answers to the questions above. You may also include additional comments for the author, including concerns about dual publication, research ethics, or publication ethics. (Please upload your review as an attachment if it exceeds 20,000 characters)

**Reviewer #2:** 1、The introduction mentions that the adverse effects of intraoperative hypothermia during open surgery include surgical site infections, coagulopathy and increased transfusion requirements, increased analgesic needs, altered drug metabolism, and adverse cardiac events. However, in the context of laparoscopic surgery, why is only arrhythmia mentioned among these adverse effects?

**Answer**: The introduction does mention the effects of intraoperative hypothermia in open surgeries as noted, but there was very little literature looking directly at the consequences of hypothermia in laparoscopic surgeries that also had inconsistent findings. Hence, the consequences of hypothermia were shown in Table 3. We also add the outcome of “intraoperative blood transfusion” into Table 3 (Lines 193-194) and S1 Table. Even though the hypothermia group was related to a higher proportion of blood transfusion requirements, it was not statistically significant in the multivariate logistic regression model. Since it is a retrospective cohort study, the surgical site infection and postoperative coagulopathy are quite limited to determine due to unwell documentation postoperatively. However, we measured the outcome that had more impact and could represent the undesirable consequences of hypothermia, such as duration of ICU admission and length of hospital stay.

2. In this study, various methods are used to monitor body temperature, and there may be errors and inconsistencies in accuracy between different methods. Could this affect the accuracy of the results? Is it necessary to conduct a subgroup analysis?

**Answer**: It is true that there are various methods used for recording temperature in our study, but the esophageal route, nasopharyngeal, and rectal temperatures all represent the core body temperature, so it should be accurate. In the case of the temperature measured from the ear (tympanic membrane), since it is less accurate with a potential error of less than 0.5 ˚C, we adjusted the recorded temperatures by adding 0.5 ˚C to correct for this discrepancy. We add the methods of temperature monitoring into Table 1. It showed a balance of methods of temperature monitoring among the hypothermia and normothermia groups (p = 0.334); therefore, the subgroup analysis among methods of temperature monitoring is not necessary. Only two patients were recorded by the rectal route, which required combination with other routes to accomplish comparison by the Chi-square test.

3. A temperature monitoring interval of every 30 minutes may be too long; this should be discussed as it represents a limitation or an area for improvement in future studies. Continuous monitoring could provide statistics on the duration of hypothermia, thereby increasing the sensitivity of the research.

**Answer**: We monitored the temperature continuously, but we only recorded every 30 minutes (S1 Fig). However, the duration of hypothermia was also measured to see the trend of the temperature change to bring more accuracy to the temperature’s data. We add this limitation into “The limitation subsection” (Lines 354-358). Longer duration of hypothermia could result in more undesirable outcomes; however, we performed different methods of warming (force air warming, warming mattress) (Lines 97-98) to raise the body’s temperature up to normal (BT ≥ 36 ˚C). during surgery. Therefore, the development of a second episode of hypothermia after temperature resumed to normal was considered an exposure to hypothermia-related outcomes/consequences of hypothermia in our laparoscopic patients.

7. PLOS authors have the option to publish the peer review history of their article ([what does this mean?](https://track.editorialmanager.com/CL0/https:%2F%2Fjournals.plos.org%2Fplosone%2Fs%2Feditorial-and-peer-review-process%23loc-peer-review-history/1/010f01931b182552-91e9c1a8-5ddb-4cd4-9eff-2c095b890a35-000000/j582zNNxs804SDiXCxoQg-v9Ea9zCgoUNsCKlmVv3xY=184)). If published, this will include your full peer review and any attached files.


If you choose “no”, your identity will remain anonymous but your review may still be made public.


**Do you want your identity to be public for this peer review?** For information about this choice, including consent withdrawal, please see our [Privacy Policy](https://track.editorialmanager.com/CL0/https:%2F%2Fwww.plos.org%2Fprivacy-policy/1/010f01931b182552-91e9c1a8-5ddb-4cd4-9eff-2c095b890a35-000000/8oKHspYxIBDwxT_Z0NM9UxF7vnKiD3EfyQI13TjPOT4=184).

Reviewer #2: No

Should you have more comments, please don’t hesitate to contact us

Thank you for your attention to our paper

Sincerely yours,

Dr. Tashi Penjore

Assoc. Prof. Maliwan Oofuvong

Assist. Prof. Sunisa Chatmongkolchart

Assist. Prof. Chanatthee Kitisiripant

Rongrung Rueangchira-urai

Jaranya Leeratiwong
